# Validating epilepsy diagnoses in routinely collected data

**DOI:** 10.1016/j.seizure.2017.10.008

**Published:** 2017-11

**Authors:** Beata Fonferko-Shadrach, Arron S. Lacey, Catharine P. White, H.W. Rob Powell, Inder M.S. Sawhney, Ronan A. Lyons, Phil E.M. Smith, Mike P. Kerr, Mark I. Rees, W. Owen Pickrell

**Affiliations:** a Wales Epilepsy Research Network, Institute of Life Science, Swansea University, Swansea, UK; b Morriston Hospital, Abertawe Bro Morgannwg University Health Board, Swansea, UK; c Neurology and Molecular Neuroscience Research Group, Institute of Life Science, Swansea University Medical School, Swansea University, Swansea, UK; d Farr Institute, Swansea University Medical School, Swansea University, Swansea, UK; e University Hospital of Wales, Cardiff and Vale University Health Board, Cardiff, UK; f Institute of Psychological Medicine and Clinical Neuroscience, Cardiff University, Cardiff, UK

**Keywords:** Diagnosis, Validation, Routinely collected data, Epilepsy

## Abstract

**Purpose:**

Anonymised, routinely-collected healthcare data is increasingly being used for epilepsy research. We validated algorithms using general practitioner (GP) primary healthcare records to identify people with epilepsy from anonymised healthcare data within the Secure Anonymised Information Linkage (SAIL) databank in Wales, UK.

**Method:**

A reference population of 150 people with definite epilepsy and 150 people without epilepsy was ascertained from hospital records and linked to records contained within SAIL (containing GP records for 2.4 million people). We used three different algorithms, using combinations of GP epilepsy diagnosis and anti-epileptic drug (AED) prescription codes, to identify the reference population.

**Results:**

Combining diagnosis and AED prescription codes had a sensitivity of 84% (95% ci 77–90) and specificity of 98% (95–100) in identifying people with epilepsy; diagnosis codes alone had a sensitivity of 86% (80–91) and a specificity of 97% (92–99); and AED prescription codes alone achieved a sensitivity of 92% (70–83) and a specificity of 73% (65–80). Using AED codes only was more accurate in children achieving a sensitivity of 88% (75–95) and specificity of 98% (88–100).

**Conclusion:**

GP epilepsy diagnosis and AED prescription codes can be confidently used to identify people with epilepsy using anonymised healthcare records in Wales, UK.

## Highlights

• Cases with and without epilepsy were linked with anonymised primary care data.
• Primary care diagnosis and drug codes accurately identify the cases with epilepsy.
• Drug codes alone can be used to identify children with epilepsy.
• Combining drug and diagnosis codes for adults and children increases accuracy.

## 1 Introduction

Vast amounts of electronic, routinely-collected, medical and related administrative data are generated in modern healthcare systems. These data can be anonymised, linked and used for healthcare research [1], [2]. Large numbers of individuals can be studied without having to specifically recruit individuals for projects, which can be expensive, time-consuming and introduce selection bias. Records can also be linked from a wide variety of different sources, enabling a wide breadth of data to be analysed. Routinely-collected data are increasingly being used for high quality epilepsy studies [3], [4], [5].

Every individual in the United Kingdom (UK) is entitled to register with a primary care General Practitioner (GP) and there is evidence that almost everyone in the UK does register with a GP [6]. GPs have a central role in providing primary care for people with epilepsy through assessment, diagnosis, appropriate referral to secondary and tertiary services, managing and prescribing medications (including the vast majority of anti-epileptic drugs) and creating and maintaining a centralised health care record. GPs are the patient’s primary contact point for access to specialist services. GP health records contain details of encounters with GPs and other healthcare providers using Read codes.

Read codes are the current clinical terminology coding system used in UK primary care systems to record symptoms, diagnosis and prescriptions [7]. Read codes are hierarchical (with increasing level of detail with increasing digits) e.g: *F25* is used to record epilepsy, *F25A*. is used for juvenile myoclonic epilepsy and *F2540* for temporal lobe epilepsy.

GP records have been used as the basis for epilepsy studies within data repositories such as the clinical practice research datalink (CPRD) and the Secure Anonymised Information Linkage (SAIL) databank [4], [8], [9]. One of the limitations of using routinely-collected data for epilepsy studies is the possibility of including incorrectly recorded epilepsy diagnoses. In particular, it’s not known how accurately epilepsy diagnoses made by hospital specialists are recorded in GP records. Guidelines advise that algorithms used for case ascertainment in routinely-collected data studies are validated in each population studied [10]. The accuracy of UK GP diagnosis codes has been validated for many diseases but, to our knowledge, has only been partially validated for epilepsy diagnosis [8], [9], [11]. In this study we specifically aimed to validate the accuracy of algorithms using GP records to identify people with epilepsy from anonymised, linked, routinely collected Welsh healthcare data.

## 2 Method

In Wales, anonymised GP primary care electronic health records are collated and linked with other data within the Secure Anonymised Information Linkage (SAIL) databank [1], [12]. We searched the SAIL databank on 13th April 2016, at this time GP records were available up to 31st December 2015 and there were records for 73% of GP practices across Wales (approx. 2.4 million people). GP records can be tracked over time, so that individual patient’s records can be analysed longitudinally through multiple GP practices. We used combinations of epilepsy diagnosis and anti-epileptic drug (AED) prescription codes to create three epilepsy case ascertainment algorithms.

We anonymously uploaded and linked a list of 150 individuals with epilepsy and 150 individuals without epilepsy (reference population) to existing SAIL records, using an established and validated split-file approach [1], [12]. We then compared the performance of the three different epilepsy case ascertainment algorithms within SAIL in identifying the reference population.

### 2.1 The reference population

The Swansea Epilepsy Database currently holds detailed clinical information (including diagnosis, medications, imaging and EEG results) for 960 patients seen by a clinician with a specialist interest in epilepsy (neurologist or paediatric neurologist) treated at Morriston Hospital, Swansea. 283 (29%) of these patients have generalised epilepsy, 510 (53%) have focal epilepsy, 125 (13%) have unclassifiable epilepsy and 42 (4%) have an uncertain diagnosis.

Between January and March 2015, we examined the database and used a random number generator to select a sample of 100 adults (50 men and 50 women, who were over 16 at their last consultation date) and 50 children (25 boys and 25 girls, who were 16 and under at their last consultation date) with a clinically definite diagnosis of epilepsy from the database. The clinical record and investigation results for each of these 150 individuals were reviewed to confirm a clinically definite diagnosis as per the International League Against Epilepsy’s (ILAE) practical clinical definition of epilepsy. These 150 individuals formed the reference population of people *with epilepsy*.

To ascertain a control cohort, 300 patients were reviewed from consecutive general neurology clinics run by neurologists and paediatric neurologists. Their diagnosis was checked using clinic letters stored in an electronic format on the hospital system. Patients with a diagnosis of epilepsy were excluded. Using a random number generator, we randomly selected a sample of 100 adults (50 men and 50 women, who were over 16 at their last consultation date) and 50 children (25 boys and 25 girls, who were 16 and under at their last consultation date) from these 300 patients. These 150 individuals formed the reference population of people *without* epilepsy.

We have previously estimated the sensitivity of an epilepsy case ascertainment algorithm at 90% using GP diagnosis and AED prescription [9]. Based on this, a sample size of 150 provides a 95% confidence interval of 10% for sensitivities (proportions) of 90%.

### 2.2 Algorithm construction and assessment

We used three different algorithms to identify people with epilepsy within SAIL: A) individuals with an epilepsy diagnosis Read code and two prescriptions of the same AED within six months; B) individuals with an epilepsy diagnosis Read code only and C) individuals with two prescriptions of the same AED within six months only.

We used version 2 Read codes. For diagnosis we used *F25* and all subcodes beginning with *F25* as well as Read codes *1O30*, *667B*., and *SC200*. For AEDs we used *dn* and *do* (and all sub codes). For a full list of Read codes used see Supporting information in Pickrell et al 2015 [9].

### 2.3 Analysis and statistical tests

True positive (TP) cases had a hospital diagnosis of epilepsy and were identified within SAIL as having epilepsy; true negative (TN) cases did not have epilepsy as confirmed by hospital records and were not identified as having epilepsy within SAIL; false positive (FP) cases did not have epilepsy as confirmed by hospital records and were identified as having epilepsy within SAIL; and false negative (FN) cases had a hospital diagnosis of epilepsy and were not identified as having epilepsy within SAIL. Positive predictive value (PPV) was defined as TP/(TP + FP); sensitivity TP/(TP + FN); specificity TN/(TN + FP) and false positive rate (FPR) as FP/(FP + TN). We calculated Youden’s index (J) using sensitivity + specificity–1, as a measure of the accuracy of the algorithms. J ranges from −1 to 1 (*J* = 1 for a perfect test) [13]. Confidence limits were calculated using the exact binomial method. We used R version 3.0.1 to perform the statistical analysis.

### 2.4 Ethical approval

This study was approved by SAIL’s independent Information Governance Review Panel (project 387). The National Research Ethics Service has confirmed that SAIL projects using anonymised data do not require specific NHS research ethics committee approval.

## 3 Results

145 of the 150 reference cases with epilepsy (97%) and 143 of the 150 reference cases without epilepsy (95%) were found to be registered with a SAIL GP. The sensitivity, specificity, positive predictive value, false positive rate and accuracy of each of the three algorithms in identifying the reference cases are shown in Table 1.

**Table 1:** Proportion of epilepsy cases (n = 145) and cases without epilepsy (n = 143) identified within SAIL using three different algorithms: A − Individuals with a primary care epilepsy diagnosis code and at least two consecutive codes for prescription of an anti-epileptic drugs (AED); B − Individuals with an epilepsy diagnosis code only; C − Individuals with at least two consecutive codes for prescription of an AED. See method section for definitions of positive predictive value, sensitivity, false positive rate, specificity and Youden’s Index.*We included 145 (97 adults, 48 children) people with a hospital diagnosis of epilepsy and 143 (98 adults and 45 children) people without a hospital diagnosis of epilepsy.

Patients within SAIL identified as having epilepsy			Hospital neurology service diagnosis of epilepsy*		Positive predictive value (95% Cl)	Sensitivity (95% CI)	False positive rate (95% CI)	Specificity (95% CI)	Youden’s Index (J)
Algorithm Used									
A − Epilepsy diagnosis & AED	All patients		Yes	No					
		Yes	122	2	98% (94–100)	84% (77–90)	1% (0–5)	99% (95–100)	0.83
		No	23	141					
	Adults	Yes	84	2	98% (92–100)	87% (78–93)	2% (0–7)	98% (93–100)	0.85
		No	13	96					
	Children	Yes	38	0	100% (91–100)	79% (65–90)	0% (0–8)	100% (92–100)	0.79
		No	10	45					
B − Epilepsy diagnosis only	All patients	Yes	125	5	96% (91–99)	86% (80–91)	3% (1–8)	97% (92–99)	0.83
		No	20	138					
	Adults	Yes	85	2	98% (92–100)	88% (80–93)	2% (0–7)	98% (93–100)	0.86
		No	12	96					
	Children	Yes	40	3	93% (81–99)	83% (70–93)	7% (1–18)	93% (82–99)	0.76
		No	8	42					
C − AED only	All patients	Yes	133	39	77% (70–83)	92% (86–96)	27% (20–35)	73% (65–80)	0.65
		No	12	104					
	Adults	Yes	91	38	71% (63–78)	94% (87–98)	39% (30–49)	61% (51–71)	0.55
		No	6	60					
	Children	Yes	42	1	98% (94–100)	88% (75–95)	2% (0–12)	98% (88–100)	0.86
		No	6	44					

## 4 Discussion

Our results show that anonymised GP records can be used to accurately identify patients with epilepsy diagnosed by a hospital specialist in Wales. The best sensitivities achieved for all patients, adults and children were 92%, 94% and 88% respectively. The corresponding figures for specificity were 99%, 98% and 100%. These figures compare well with sensitivities and specificities from other similar epilepsy case definition validation studies in different healthcare systems e.g. Australian, Italian and American studies achieved sensitivities of 82–90% and specificities of 94–100% [14], [15], [16]. We have previously used similar algorithms within the SAIL databank to estimate the prevalence of epilepsy in Wales to be 0.77% (95% CI 0.76–0.79%) [9].

As with previous studies, algorithm A (diagnosis and AED code) is the most specific (98–100%) given that it has the ‘narrowest’ criteria and algorithm C (AED only) is the most sensitive (88–94%) with its ‘broader’ criteria. The large difference in specificity between adults and children for algorithm C (61% c.f. 98%) can be explained by the widespread use of AEDs for indications other than epilepsy in adults (e.g. migraine, mood disorders and neuropathic pain). AEDs are seldom prescribed for indications other than epilepsy in children in the UK [8]. Our results suggest that using criteria of AED prescription alone can be used to identify children with epilepsy.

There was surprisingly little difference in performance between algorithm A and B, Algorithm A (additional AED code) was more specific than algorithm B and algorithm B was more sensitive but their overall accuracy was comparable. GP diagnosis codes for epilepsy therefore seem reliable in their own right. Although this is expected, given that epilepsy diagnosis should be made in secondary care in the UK and later transcribed into the primary care record by GPs [17], to our knowledge this has not been described before and is an important result for future research involving GP epilepsy diagnosis codes.

Several factors may have improved UK GP epilepsy diagnosis coding practice in recent years. The Quality Outcomes Framework (QOF) for GPs was introduced in 2004 and provides financial incentives to GPs who achieve certain indicators. The current QOF indicator for epilepsy includes a record of patients aged 18 or over on drug treatment for epilepsy who have been seizure-free for the last 12 months [18]. Previous QOF indicators have included an indicator for maintaining a register of adults with epilepsy on anti-epileptic drug treatment. Current UK guidelines for the diagnosis and management of epilepsy advise structured management systems and regular reviews within primary care which are likely to encourage accurate epilepsy diagnosis coding [17], [19].

We have used relatively small numbers in the reference population in this study due to the resources needed to manually check medical records and test results. We could not review the anonymised data within SAIL to ascertain the reasons for the false positive and false negative cases. We also did not stratify the non-epilepsy cases into epilepsy mimics such as dissociative seizures and acute symptomatic seizures which may have a higher miscoding rate in GP records. At the time of analysis, 73% of the Welsh population’s GP records were available within SAIL. This lack of 100% coverage likely explains why a small number of the reference cases with and without epilepsy were not ‘found’ in SAIL.

These results are specific to primary care records in Wales and are not applicable to other healthcare systems or methods of ascertaining epilepsy cases (for example hospital discharge summaries). Other parts of the UK do have similar healthcare systems and although the results may be generalizable to the remainder of the UK further work needs to be done to prove this. Currently there is no facility to include EEG and imaging data within SAIL and so we could not include these in our ascertainment algorithms. Additionally it is impossible to identify people with epilepsy who do not attend their GP or have not been seen by a hospital specialist.

Our reference epilepsy cohort was obtained from a secondary care epilepsy database which may have provided a bias towards people with more severe epilepsy. We selected a group of people without epilepsy from patients who had attended general neurology clinics as a control group. This group therefore does not represent the ‘general’ population without epilepsy. However, this group of patients may be considered as a ‘better test’ of ascertainment algorithms as patients with other neurological conditions may be more likely to be incorrectly coded as having epilepsy than the general population. Conversely it is also possible (although unlikely in our opinion) that neurologists would not record a diagnosis of epilepsy in a general neurology clinic appointment with a different focus (e.g. headache).

The strengths of our study are that we have used a carefully validated reference population with an epilepsy diagnosis from an epilepsy specialist and without an epilepsy diagnosis to validate algorithms within an established anonymised databank containing extensive primary care records for at least 2.4 million people.

## 5 Conclusion

Using primary care epilepsy diagnosis codes is an accurate method to identify patients with epilepsy within the SAIL databank. Using AED prescription codes in addition to epilepsy diagnosis codes increases the specificity and positive predictive value by 2% at a cost of a 2% reduction in sensitivity. In children using AED prescription codes alone is an accurate way to identify epilepsy cases. These results are generalizable to other studies that use UK primary care records for epilepsy case ascertainment and can serve as a baseline measure of accuracy of case ascertainment in such studies.

## Disclosure of conflicts of interest

Author MK has received honoraria from UCB and Johnson and Johnson. The remaining authors have no conflicts of interest. We confirm that we have read the Journal’s position on issues involved in ethical publication and affirm that this report is consistent with those guidelines.
